# Turning the Tide: Natural Products and Natural-Product-Inspired Chemicals as Potential Counters to SARS-CoV-2 Infection

**DOI:** 10.3389/fphar.2020.01013

**Published:** 2020-07-02

**Authors:** Zhonglei Wang, Liyan Yang

**Affiliations:** ^1^ School of Chemistry and Chemical Engineering, Qufu Normal University, Qufu, China; ^2^ School of Pharmaceutical Sciences, Tsinghua University, Beijing, China; ^3^ School of Physics and Engineering, Qufu Normal University, Qufu, China

**Keywords:** COVID-19, SARS-Cov-2, natural products, natural-product-inspired, potential anti-SARSCoV-2 agents

## Abstract

The novel and highly pathogenic severe acute respiratory syndrome coronavirus 2 (SARS-CoV-2), which causes coronavirus disease 2019 (COVID-19), has become a continued focus of global attention due to the serious threat it poses to public health. There are no specific drugs available to combat SARS-CoV-2 infection. Natural products (carolacton, homoharringtonine, emetine, and cepharanthine) and natural product-inspired small molecules (ivermectin, GS-5734, EIDD-2801, and ebselen) are potential anti-SARS-CoV-2 agents that have attracted significant attention due to their broad-spectrum antiviral activities. Here, we review the research on potential landmark anti-SARS-CoV-2 agents, systematically discussing the importance of natural products and natural-product-inspired small molecules in the research and development of safe and effective antiviral agents.

## Introduction

Coronavirus disease 2019 (COVID-19), caused by severe acute respiratory syndrome coronavirus 2 (SARS-CoV-2), continues to be the subject of global attention due to the serious threat it poses to public health (Wang et al., 2020; Zhu et al., 2020). Currently, there are no specific drugs available to combat SARS-CoV-2 (Kupferschmidt and Cohen, 2020). The epidemic is ongoing and, as of 12 June, 2020, the World Health Organization (WHO) reported that there had been 7,410,510 confirmed cases worldwide, including 418,294 deaths (Coronavirus disease (COVID-2019)).

This means that more aggressive trials of drugs to prevent and treat SARS-CoV-2 infection should be intensely pursued across the globe (Kalil, 2020). Therefore, there is an urgent need to identify agents for treating SARS-CoV-2 infection (Li and De Clercq, 2020). As a major source for drugs and drug leads, natural products and natural-product-inspired agents have attracted significant attention, and they have played an integral role in the treatment of many different conditions (Newman and Cragg, 2020). Since the start of the multinational COVID-19 outbreak, significant progress has been made in identifying natural products and natural-product-inspired small molecules that may serve as anti-SARS-CoV-2 drugs. This review systematically discusses the current progress regarding potential anti-SARS-CoV-2 natural products and natural-product-inspired small molecules.

## Promising Natural Products for Treating SARS-CoV-2 Infection

Natural products possess tremendous structural diversity and unique chemical diversity, and they continue to serve as excellent starting points for inspiring new drug discovery (Shen, 2015). The history of the modern pharmaceutical industry includes many stories about how natural products profoundly inspired drug discovery (Li and Weng, 2017). With the current technological advances, natural products remain potentially transformative drugs for many health conditions. The growing understanding of efficient antiviral drug development has led to the exploration of natural products as an important tactic for identifying effective COVID-19 treatments.

**Figure 1:**
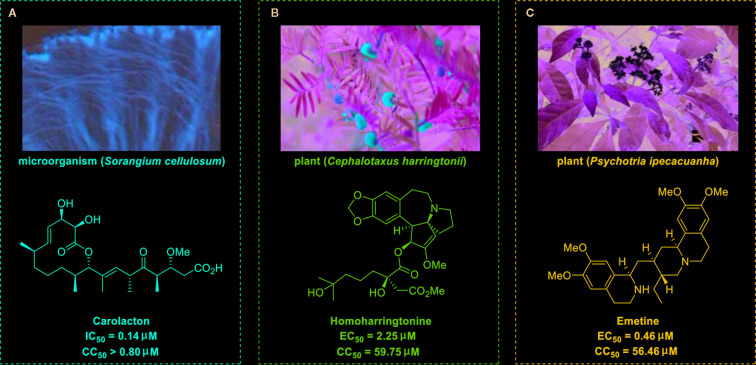
Promising natural products for treating COVID-19. **(A)** Carolacton was isolated from the myxobacterium *Sorangium cellulosum*. **(B)** Homoharringtonine was isolated from the plant *Cephalotaxus harringtonii*. **(C)** Emetine was isolated from the plant *Psychotria ipecacuanha*.

Carolacton, produced by the myxobacterium *Sorangium cellulosum*, is an antibacterial macrolide keto-carboxylic acid (**Figure 1A**) (Jansen et al., 2010). *In vitro*, it demonstrates significant bioactivity against the human pathogen *Streptococcus mutans* by reducing the number of viable cells in biofilms (Fu et al., 2020), and it has been found to be a methylenetetrahydrofolate dehydrogenase 1 (MTHFD1) inhibitor (Anderson et al., 2020). SARS-CoV-2 may have originated in bats (Shi et al., 2020) (as a study has shown that its genome is similar to that of the bat coronavirus RaTG13, with 96.2% identity (Zhou et al., 2020)) and, very recently, Tan’s group demonstrated that MTHFD1 is a critical host factor for the viral RNA replication of a broad spectrum of viruses in both bats and humans (Anderson et al., 2020). Based on in-depth research, Tan’s group demonstrated that MTHFD1 is a potential target for developing anti-SARS-CoV-2 agents and that the MTHFD1 inhibitor carolacton strongly inhibited SARS-CoV-2 replication in Vero cells at a half maximal inhibitory concentration (IC_50_) of as low as 0.14 μM and a moderate cytotoxicity (50% cytotoxic concentration [CC_50_] >0.80 μM) (Anderson et al., 2020). Thus, there is hope that this antimicrobial natural product may be useful in the COVID-19 epidemic.

**Figure 2:**
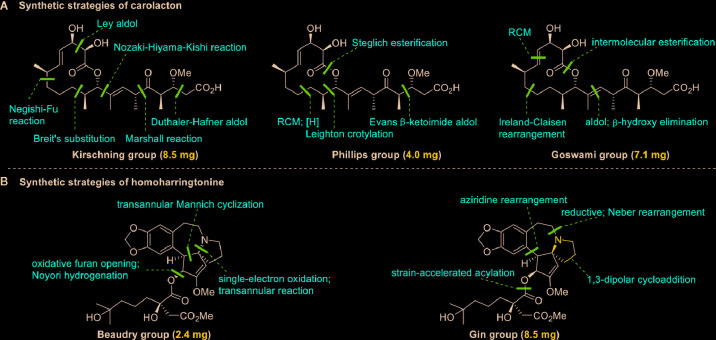
Key strategies in the synthesis of carolacton and homoharringtonine. **(A)** Synthetic strategies of carolacton by Kirschning’s group, Phillips’s group and Goswami’s group, respectively. **(B)** Synthetic strategies of homoharringtonine by Gin’s group and Beaudry’s group, respectively.

Regarding the total synthesis of carolacton, three examples of milligram-scale processes have been reported. In 2012, Kirschning’s group (Schmidt and Kirschning, 2012) reported on the first total synthesis of carolacton (with an overall yield of 4.3%) on an 8.5-mg scale in 22 linear steps. The key transformations involved asymmetric Nozaki-Hiyama-Kishi cross-coupling and Negishi-Fu coupling as well as the metal-mediated Ley aldol reaction, Duthaler-Hafner aldol reaction, Marshall reaction, and Breit’s substitution. Two years later, Phillips’s group and Wuest’s group (Hallside et al., 2014) collaboratively developed an efficient synthesis of carolacton (with an overall yield of 7.9%) on a 4.0-mg scale in just 14 linear steps. This synthesis process involved ring-closing metathesis (RCM), selective reduction, Leighton crotylation, and Steglich esterification. In 2017, Goswami’s group (Kuilya and Goswami, 2017) reported a third total synthesis (with an impressive overall yield of 18.8%) on a 7.1-mg scale in just 13 linear steps. The key strategies included Urpi acetal aldol reaction, β-hydroxy elimination, intermolecular esterification, and RCM. The three elegant synthesis strategies for producing carolacton are shown in **Figure 2A**. However, in the current research and clinical contexts, the development of a ton-scale process to synthesize carolacton is urgently needed.

Homoharringtonine (omacetaxine) is a natural product that was isolated from the plant *Cephalotaxus harringtonii* in 1970 (Powell et al., 1970). It was approved by the US Food and Drug Administration (FDA) in 2012 as an effective anti-cancer agent to treat chronic myeloid leukemia (**Figure 1B**) (Mullard, 2013). Additionally, it exhibits broad-spectrum activities against viruses, such as mouse hepatitis virus (MHV) at an IC_50_ of 0.012 μM (Cao et al., 2015), herpes simplex virus type 1 (HSV-1) at an IC_50_ of 0.14 μM (Dong et al., 2018), foot and mouth disease virus (FMDV) at an IC_50_ of 3.05 μM (Gong et al., 2019), and echovirus 1 (EV1) at a half maximal effective concentration (EC_50_) of 0.12 μM (Andersen et al., 2019). Two elegant milligram-scale total syntheses of homoharringtonine have been reported by Gin’s group (Eckelbarger et al., 2008) and Beaudry’s group (Ju and Beaudry, 2019), respectively (**Figure 2B**). In 2008, Gin’s group reported a novel total synthesis of homoharringtonine on an 8.5-mg scale, using aziridine rearrangement and 1,3-dipolar cycloaddition as key strategies. In 2019, Beaudry’s group developed an excellent total synthesis of homoharringtonine involving 12 linear steps on a 2.4-mg scale. The key strategies were oxidative furan ring opening with spontaneous transannular Mannich reaction as well as the Noyori hydrogenation reaction.

The tetrahydroisoquinoline alkaloid emetine is an older natural product that has potential cardiotoxicity and was isolated from the plant *Psychotria ipecacuanha* (**Figure 1C**) (Wiegrebe et al., 1984). As a protein synthesis inhibitor, emetine has been widely used in pharmacology (Akinboye et al., 2012). Furthermore, emetine has recently been recognized as a promising broad-spectrum antiviral drug with *in vitro* activity against multiple viruses, including MHV-A59 at an EC_50_ of 0.12 μM (Shen et al., 2019), severe acute respiratory syndrome coronavirus (SARS-CoV) at an EC_50_ of 0.051 μM (Dyall et al., 2014), Middle East respiratory syndrome coronavirus (MERS-CoV) at an EC_50_ of 0.014 μM (Dyall et al., 2014), and Ebola virus (EBOV) at an IC_50_ of 0.017 μM (Yang et al., 2018). Very recently, Peterson’s group showed that emetine concentrations could be 300 times higher in the lungs compared to in the blood, and emetine may achieve therapeutic concentrations at viral infection sites, especially in the lungs (Bleasel and Peterson, 2020).

Yen’s group recently revealed that both homoharringtonine and emetine could effectively inhibit the replication of SARS-CoV-2 in Vero E6 cells at an EC_50_ of 2.55 and 0.46 μM, respectively (Choy et al., 2020). Additionally, the combination of emetine and the C-nucleoside analog GS-5734 exhibited a synergistic inhibitory effect against SARS-CoV-2 replication (Choy et al., 2020). Multiple lines of evidence have shown the potential usefulness of homoharringtonine and emetine as treatments for viral infections, but further research is needed to explore whether they exhibit anti-SARS-CoV-2 activity *in vivo*.

**Figure 3:**
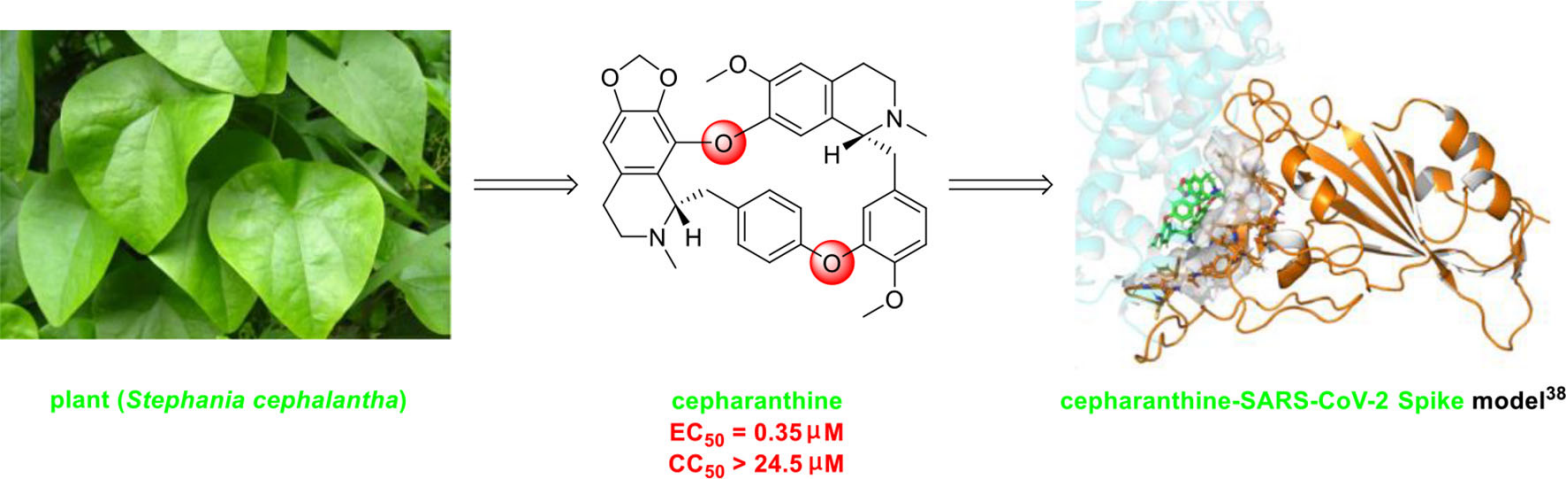
Promising natural product cepharanthine for treating COVID-19 (image reproduced from ref. 38, bioRxiv, doi: 10.1101/2020.04.14.039925).

The bisbenzylisoquinoline alkaloid cepharanthine is a natural product isolated from the plant *Stephania cephalantha*, which is used as a traditional herbal medicine (**Figure 3**) (Bailly, 2019). Specifically, this approved drug has significant bioactivity against several diseases, with anti-viral, anti-malarial, and anti-cancer effects (Fang et al., 2013). It has IC_50_ values of 0.026 μM and 9.5 μg/mL against HIV-1 (Okamoto et al., 1998) and SARS-CoV (Zhang et al., 2005), respectively. Furthermore, it dramatically blocked the viral replication in human coronavirus OC43 (HCoV-OC43)-infected MRC-5 human lung cells (with an IC_50_ of 0.83 μM) and inhibited viral S and N protein expression (Kim et al., 2019). Watashi’s group recently revealed that cepharanthine could effectively inhibit SARS-CoV-2 replication *in vitro* with an EC_50_ at 0.35 μM with minimal toxicity (selectivity index >70) (Ohashi et al., 2020). It is worth noting that cepharanthine is an approved drug with a good safety profile, highlighting a new potential role for cepharanthine regarding inhibiting SARS-CoV-2 replication.

There are currently no data on drug–drug interactions involving the abovementioned natural products (The liverpool drug interaction group), and close attention should be paid to these interactions during therapeutic use because drug–drug interactions will play significant roles in the safety and effectiveness of anti-COVID-19 agents.

## Promising Natural Product-Inspired Agents for Treating SARS-CoV-2 Infection

**Figure 4:**
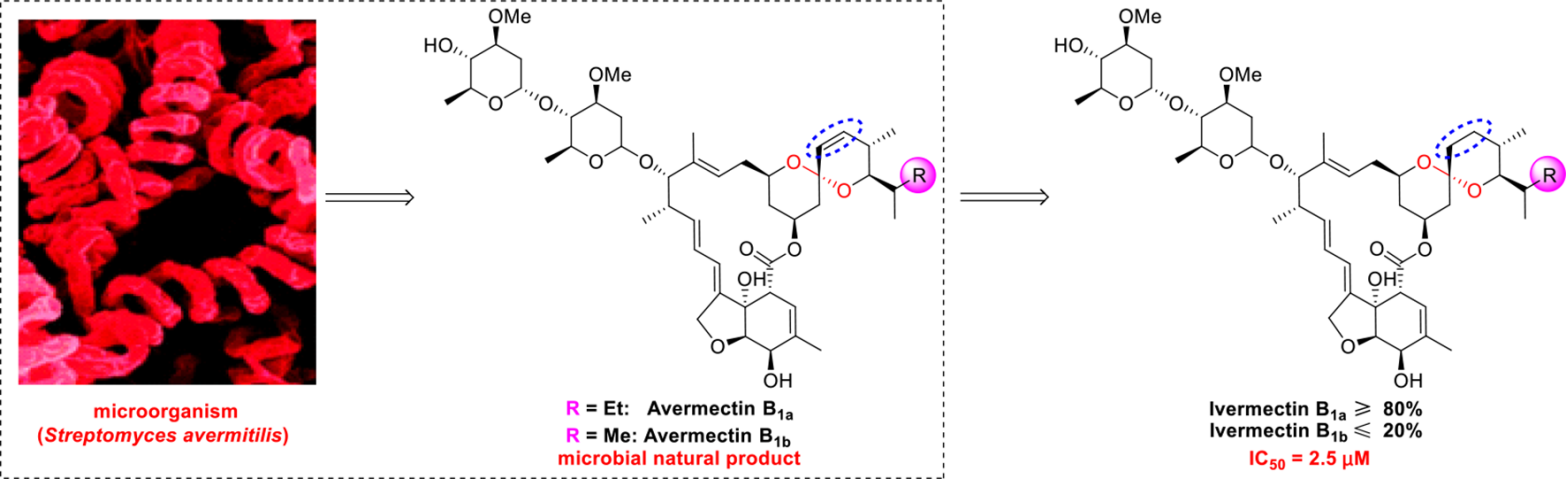
Promising natural-product-inspired ivermectin for treating COVID-19.

Ivermectin (brand name: Stromectol) is a landmark broad-spectrum anti-parasitic drug that was developed by Ōmura’s group along with Merck Sharp and Dohme Research Laboratories in 1978 (Burg et al., 1979). It has been demonstrated to be highly effective (94.9% efficacy at 24 hours and 73.8% efficacy at 2 weeks) as an oral drug for the treatment of head lice (Pariser et al., 2012). Ivermectin is the semisynthetic 22,23-dihydro derivative of the natural product avermectin B_1_ (B_1a_ and B_1b_), which is produced by *Streptomyces avermitilis* (**Figure 4**) (Campbell et al., 1983). Ivermectin is one of the most widely used antibiotics for both animals and humans, and the researchers who discovered it received the Nobel Prize in Physiology or Medicine in 2015 (Campbell, 2016).

The use of ivermectin is currently being expanded. For example, Wagstaff’s group highlighted that ivermectin is highly effective at controlling SARS-CoV-2 RNA replication *in vitro*, with a 5000-fold reduction in the virus within 48 hours (Caly et al., 2020). Recently, Lohmer’s group found that a single oral dose of ivermectin is unlikely to reach the IC_50_ (2.5 μM) in the lungs (predicted lung concentration: 0.0857 μM), and they suggested that combination therapy or inhaled treatment (to increase the concentration in the lungs) could be considered as potential solutions (Schmith et al., 2020). Nevertheless, its safety in humans has been continuously documented (Buonfrate et al., 2019), and it is hoped that it will become a key component of COVID-19 treatment regimens.

**Figure 5:**
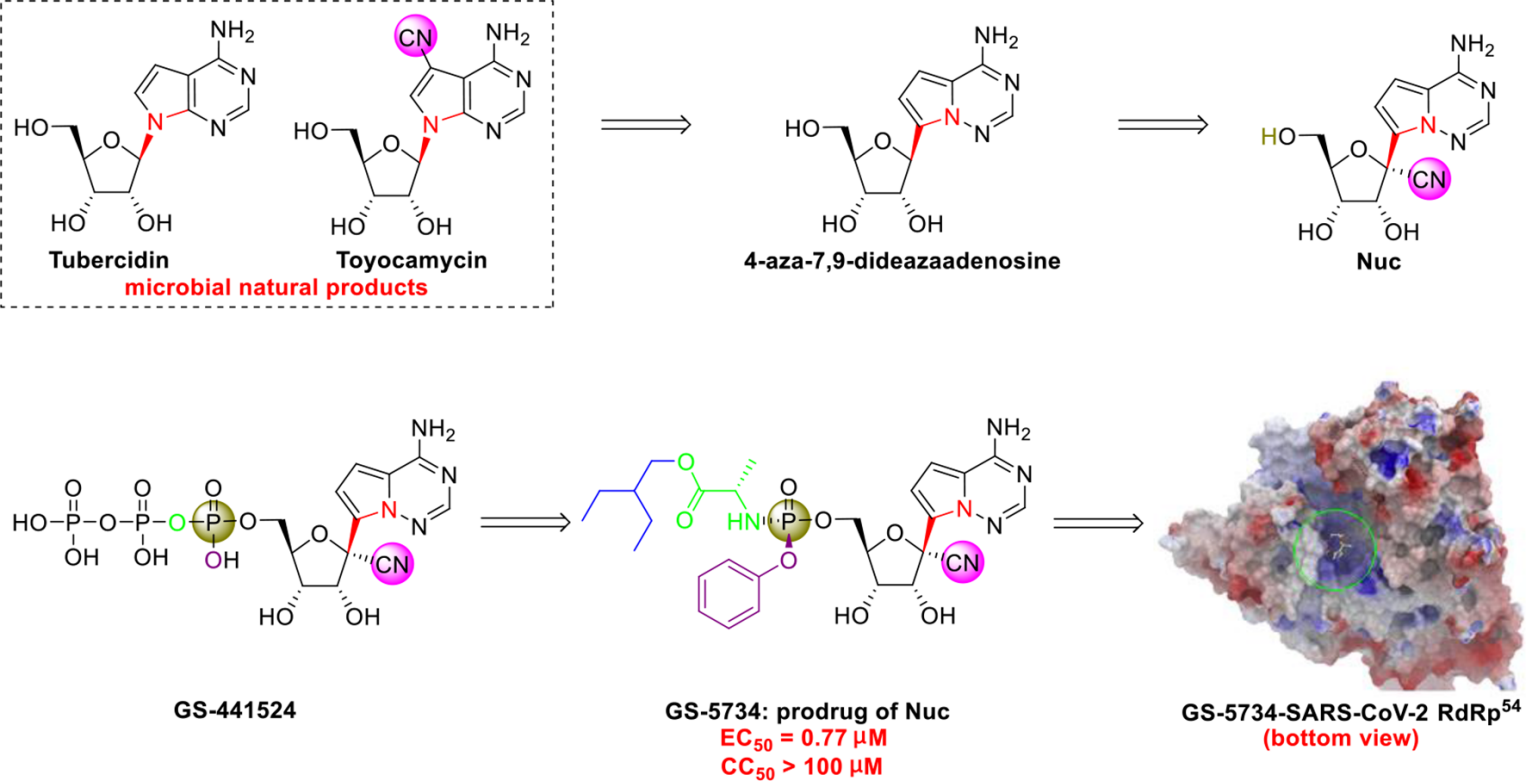
Promising natural-product-inspired GS-5734 for treating COVID-19 (image reproduced with permission from ref. 54, Acta Pharm. Sin. B, 2020, 10, 766-788).

The C-nucleoside analog GS-5734 (remdesivir), a broad-spectrum antiviral agent developed by Gilead Sciences (Warren et al., 2016), exhibited promising clinical efficacy in the treatment of the first US case of SARS-CoV-2 infection (Holshue et al., 2020). Initial research on synthesizing GS-5734 (which is a prodrug) began with structural modification of tubercidin (an antibiotic and adenosine analog that is isolated from *Streptomyces tubercidicus*) by replacing the C-N linkage with a C-C bond to create 4-aza-7,9-dideazaadenosine (**Figure 5**) (Patil et al., 1994). 4-aza-7,9-dideazaadenosine has equal cytotoxicity against HL-60 cells to tubercidin (50% infectious does [ID_50_] = 0.82 nM) and increased hydrolytic stability (Patil et al., 1994).

However, an important contribution to antiviral drug design is 1’-CN substituted Nuc, which can be viewed as a new structure that was inspired by the natural cyanide toyocamycin (isolated from *Streptomyces toyocmnsis*) (Nishimura et al., 1956). Nuc strongly inhibits hepatitis C virus *in vitro* (EC_50_ = 4.1 μM) (Cho et al., 2012). The active form of Nuc in virus-infected cells has been reported to be GS-441524, but the monophosphate conversion of Nuc to GS-441524 is a rate-limiting step (Warren et al., 2016). To overcome this issue, the monophosphorylated prodrug GS-5734 was developed to achieve better cellular uptake *in vivo* (Warren et al., 2016).

GS-5734 has been recognized as a promising broad-spectrum antiviral drug against multiple viruses including SARS-CoV (EC_50_ = 69 nM) (Sheahan et al., 2017) and MERS-CoV (EC_50_ = 20 nM) (Sheahan et al., 2017). Furthermore, Xiao’s group (Wang et al., 2020) and Yen’s group (Choy et al., 2020) recently revealed that GS-5734 is highly effective against SARS-CoV-2 infection *in vitro* (EC_50_ = 0.77 μM, viral load fitted in linear scale; or EC_50_ = 23.15 μM, viral load fitted in logarithmic scale) and has low toxicity (selectivity index >130). Regarding the mechanism of action, Li’s group highlighted that GS-5734 can bind to the RNA-binding channel of the SARS-CoV-2 RNA-dependent RNA polymerase (Wu et al., 2020) (**Figure 5**). The 1’-ribose CN substitution observed in GS-5734 plays an important role in inhibiting the viral RNA replication of SARS-CoV-2 (Zhang et al., 2020).

**Figure 6:**
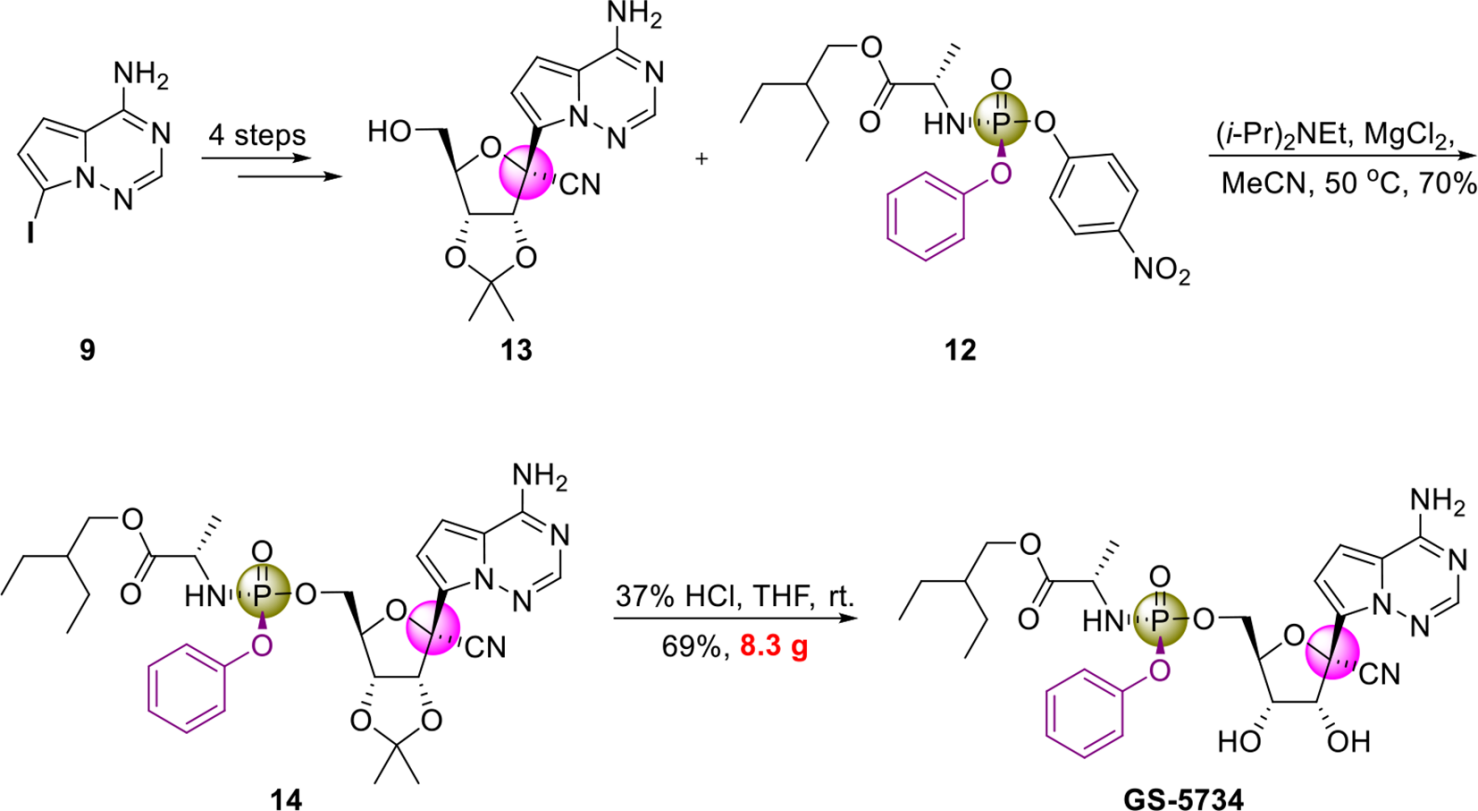
Gram-scale synthesis of GS-5734.

Notably, since 2016, GS-5734 has been reported to be safe and exhibit clinical efficacy against EBOV infection (Jacobs et al., 2016; Dörnemann et al., 2017) and SARS-CoV-2 infection (Beigel et al., 2020; Holshue et al., 2020). On April 10, 2020, Gilead Sciences reported that 68% of patients with severe COVID-19 who were treated with GS-5734 (via the compassionate use program) exhibited clinical improvement, and no new safety issues were detected (Grein et al., 2020). In addition, in May, 2020, the US FDA issued an emergency use authorization (EUA) for GS-5734 for treating SARS-CoV-2 infection (Mullard, 2020). To increase the efficiency of pharmaceutical research on GS-5734, researchers at Gilead Sciences developed a scalable process for synthesizing GS-5734 (Warren et al., 2016) (**Figure 6**). Currently, there are no drug–drug interaction data on GS-5734 (Summary on compassionate use remdesivir Gilead), but the potential for clinically significant interactions is low (The liverpool drug interaction group). It is hoped that GS-5734 will be confirmed to be a safe and effective drug against SARS-CoV-2.

**Figure 7:**
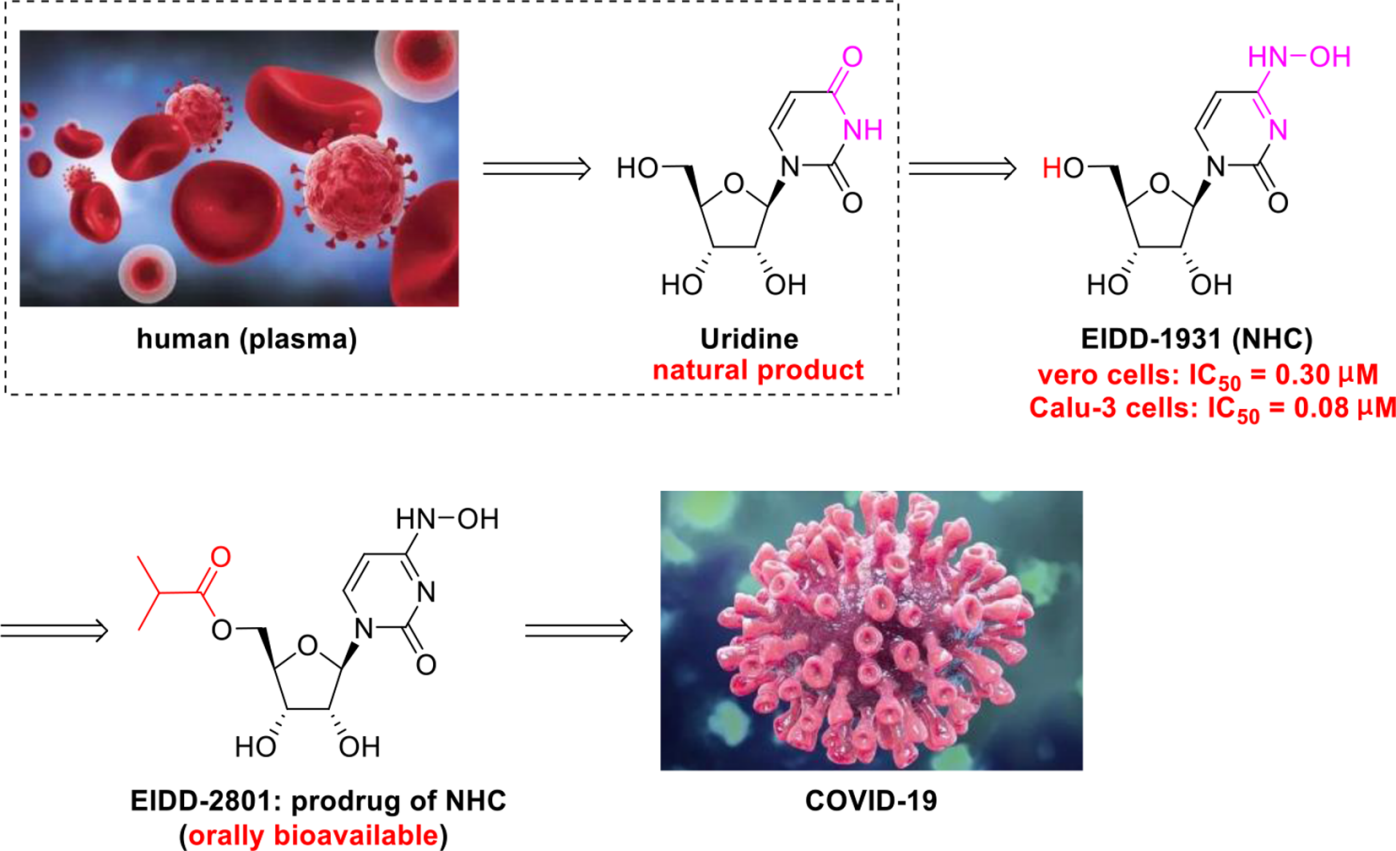
Promising natural-product-inspired EIDD-2801 for treating COVID-19.

The N-nucleoside analog EIDD-2801, a promising orally bioavailable antiviral agent, was discovered by Plemper’s group at Emory University (Toots et al., 2019). Initial research on the synthesis of EIDD-2801 began with structurally modifying the broad-spectrum antiviral agent *N* ^4^-hydroxycytidine (NHC, EIDD-1931) (Agostini et al., 2019), which was in turn derived from the essential natural product uridine found in human plasma (**Figure 7**) (Yamamoto et al., 2011).

**Figure 8:**
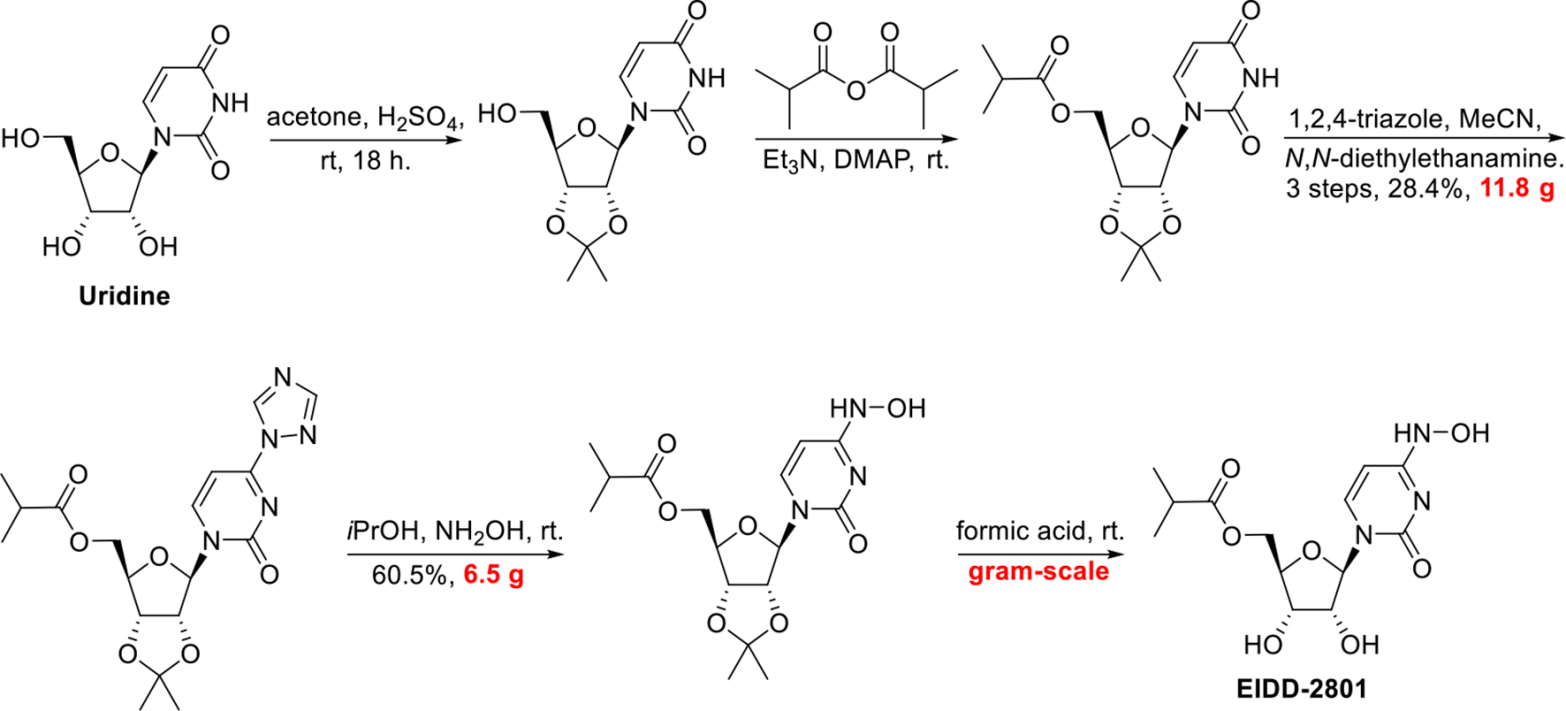
Gram-scale synthesis of EIDD-2801.

More recently, researchers at Emory University reported a scalable process for synthesizing EIDD-2801 (Painter et al., 2019) (**Figure 8**). There is currently widespread interest in the use of EIDD-2801 as a promising anti-COVID-19 agent (Rothan and Byrareddy, 2020). Baric’s group (Cho et al., 2012) highlighted that EIDD-1931 is very effective at controlling SARS-CoV-2 replication. EIDD-1931 exhibited potent anti-SARS-CoV-2 activity in Calu-3 cells (IC_50_ = 0.08 μM) and Vero cells (IC_50_ =0.30 μM), with no observable cytotoxicity at doses of up to 5 μM (Rothan and Byrareddy, 2020). Furthermore, EIDD-2801 is an orally bioavailable prodrug that is efficiently hydrolyzed *in vivo* and it exhibits remarkable selectivity (therapeutic window >1713) (Sheahan et al., 2020).

In addition, Baric’s group showed that EIDD-2801 significantly improved the pulmonary function of mice infected with MERS-CoV or SARS-CoV (Sheahan et al., 2020). Thus, EIDD-2801 has demonstrated potential effectiveness, even though there is a lack of good evidence in humans. It is hoped that researchers will work to overcome the obstacles (such as produced on a manufacturing scale, efficacy and safety *in vivo*) to allow it to be assessed in clinical research.

## Other Small Molecules With *In Vitro* Activity Against SARS-Cov-2

Growing understanding of efficient antiviral drug development has led to the use of small molecules being recognized as an important potential tactic for treating COVID-19. Rao’s group (Jin et al., 2020) showed that the organoselenium compound ebselen (**Table 1**) and other inhibitors of the SARS-CoV-2 main protease (M^pro^) exhibited potent activities, with IC_50_ values at micromolar or sub-micromolar levels (0.67–21.4 μM). Ebselen is highly effective at inhibiting SARS-CoV-2 infection (EC_50_ = 4.67 μM) and has low toxicity (median lethal dose [LD_50_] in rats >4,600 mg/kg). Most importantly, its safety in humans has been continuously evaluated in multiple clinical trials (Kil et al., 2020). Besides the abovementioned small molecules, several other natural products and natural product-inspired potential small molecules have also exhibited notable anti-SARS-CoV-2 activities (**Table 1**).

**Table 1:** Other small molecules with *in vitro* activity against SARS-Cov-2.

No.	Name	Structure	EC_50_ or IC_50_ (μM)	SI	Ref.
1	Abiraterone acetate	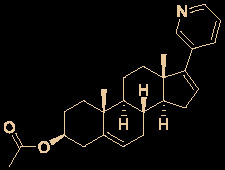	1.94	47.6	(Yuan et al., 2020)
2	ALLM	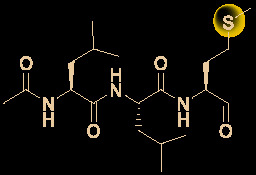	2.07	48.3	(Ma et al., 2020)
3	Amodiaquine	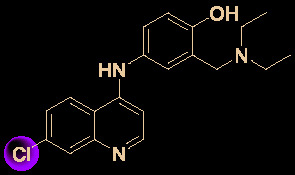	4.20	>7.1	(Ianevski et al., 2020)
4	Auranofin	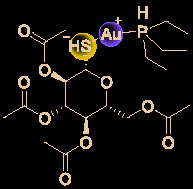	1.40	4.1	(Rothan et al., 2020)
5	Azithromycin	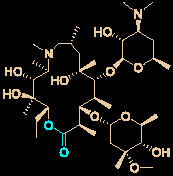	2.12	19.0	(Touret et al., 2020)
6	Baicalein	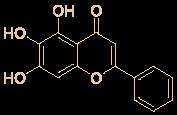	12.5-25.0	2.0	(Liu et al., 2020)
7	Baicalin	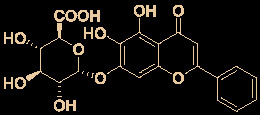	10.27	19.0	(Su et al., 2020)
8	Boceprevir	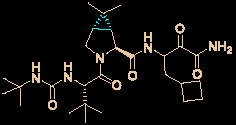	1.90	52.6	(Ma et al., 2020)
9	Carmofur	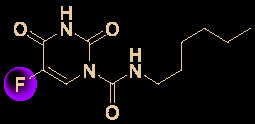	24.30	5.4	(Jin et al., 2020)
10	Cinanserin	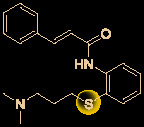	20.61	9.7	(Jin et al., 2020)
11	CVL218	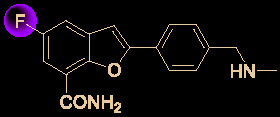	5.12	17.8	(Ge et al., 2020)
12	Digitoxin	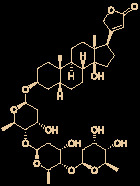	0.23	214.1	(Jeon et al., 2020)
13	Digoxin	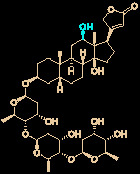	0.19	256.6	(Jeon et al., 2020)
14	Diiodohydroxyquinoline	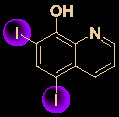	1.38	>72.5	(Ianevski et al., 2020)
15	Ebselen	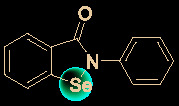	4.67	–	(Jin et al., 2020)
16	Fluspirilene	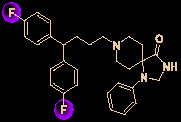	3.16	9.6	(Weston et al., 2020)
17	GC-376	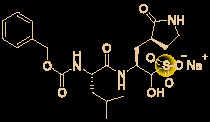	3.37	29.7	(Ma et al., 2020)
18	Hexachlorophene	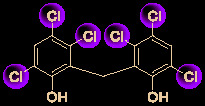	0.90	21.6	(Jeon et al., 2020)
19	MDL28170	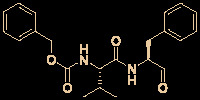	0.49	204.0	(Ma et al., 2020)
20	Nafamostat	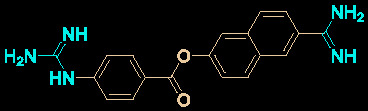	0.0022	11363	(Ko et al., 2020)
21	Nelfinavir	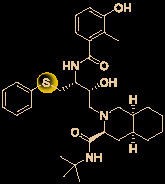	0.77	83.1	(Ohashi et al., 2020)
22	Niclosamide	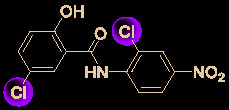	0.28	176.7	(Jeon et al., 2020)
23	Ouabain	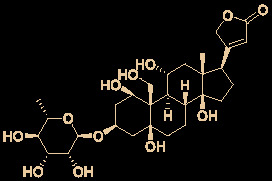	0.097	515.5	(Jeon et al., 2020)
24	Salinomycin sodium	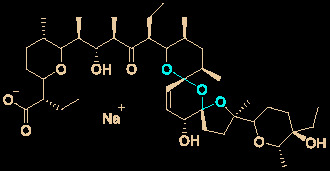	0.24	211.0	(Jeon et al., 2020)
25	S312	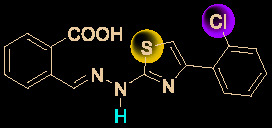	1.55	>64.6	(Xiong et al., 2020)
26	S416	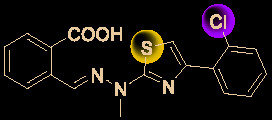	0.017	>5882	(Xiong et al., 2020)

**Figure 9:**
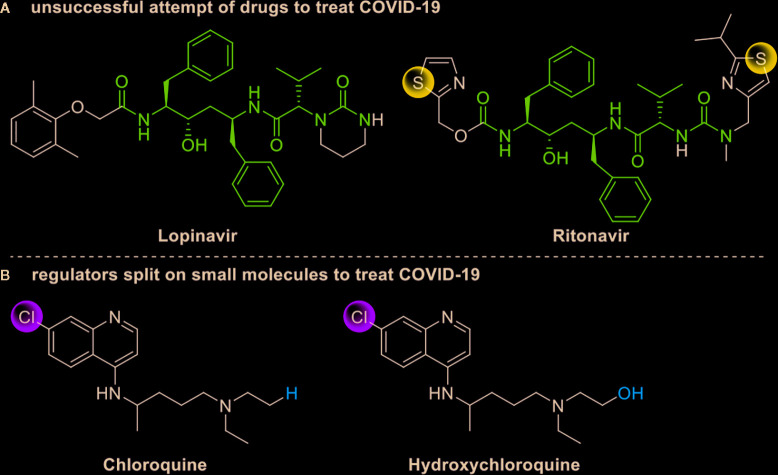
Other small molecules for treating COVID-19. **(A)** Unsuccessful attempt of lopinavir and ritonavir to treat COVID-19. **(B)** Regulators split on chloroquine and hydroxychloroquine to treat COVID-19.

Drug development carries high risks. For example, although the approved protease inhibitors lopinavir and ritonavir (**Figure 9A**) were thought to be potentially effective against SARS-Cov-2 (as they have been reported to be active against SARS^6^), Wang’s group showed that lopinavir combined with ritonavir does not seem to be highly effective in patients with COVID-19 (Cao et al., 2020).

Chloroquine and hydroxychloroquine (**Figure 9B**) have garnered considerable attention due to their ability to effectively inhibit SARS-CoV-2 (Yamamoto et al., 2011; Liu et al., 2020; Yao et al., 2020). For example, Xiao’s group (Wu et al., 2020) showed that chloroquine potently blocked SARS-CoV-2 infection at a low concentration *in vitro* (EC_50_ = 1.1 μM). Although the US FDA has authorized the use of chloroquine and hydroxychloroquine, the WHO has stated that the clinical data do not support their clinical use in COVID-19 patients (Jaffe, 2020; Taccone et al., 2020). In addition, chloroquine can be lethal (narrow therapeutic window) in children, and caution is warranted when it is used for critical illness (Smit et al., 2020).

## Conclusion and Outlook

Currently, global attention continues to be focused on COVID-19 due to its serious threat to public health. Scientists have discovered that SARS-CoV-2 has developed mutations (in 149 sites from the 103 sequenced SARS-Cov-2 strain) that have substantially changed its pathogenicity (Tang et al., 2020; Yao et al., 2020). Therefore, rapid discovery of safe, effective, and broad-spectrum anti-COVID-19 drugs is urgent. However, it is well-known that the development of a new drug usually takes more than 10 years (Ashburn and Thor, 2004). Very recently, potential anti-SARS-CoV-2 natural products and natural product-inspired small molecules have attracted significant attention due to their broad-spectrum antiviral activities. Here, we reviewed the research on potential landmark anti-SARS-CoV-2 natural products (carolacton, homoharringtonine, cepharanthine, and emetine) and natural product-inspired small molecules (ivermectin, GS-5734, EIDD-2801, and ebselen). In-depth research on potential anti-COVID-19 natural product-inspired small molecules has led to the development of multiple lines of evidence demonstrating their effects on SARS-CoV-2 infection (Ferner and Aronson, 2020; Pruijssers et al., 2020; Toots et al., 2020; Williamson et al., 2020; Xing et al., 2020).

While the current COVID-19 pandemic has led to more rapid natural product-based drug discovery and development, it is also worth noting that ton-scale total synthesis strategies for the abovementioned potential anti-SARS-CoV-2 natural products (such as carolacton and homoharringtonine) are urgently needed. However, significant challenges (for example, attainment of clinical evidence regarding the anti-SARS-CoV-2 effects of the agents in patients, and traditional drug development approach in SARS-CoV-2 is becoming untenable) will need to be overcome in order for successful clinical research to be completed. We hope that natural products and natural product-inspired small molecules will be shown to be safe and effective for treating SARS-CoV-2 infection.

## Author’s Note

Figures were reproduced with permission through Copyright Clearance Center’s RightsLink^®^ service.

## Author Contributions

ZW conceived the review. LY collected the literatures. ZW, and LY wrote the manuscript. ZW edited the manuscript. All authors read and approved the final version of the manuscript.

## Funding

Project supported by the PhD research start-up funds of Qufu Normal University, China (Grant Nos. bsqd20190164, and bsqd20190060) and the project of introduction and cultivation for young innovation talents in colleges and universities of Shandong Province.

## Conflict of Interest

The authors declare that the research was conducted in the absence of any commercial or financial relationships that could be construed as a potential conflict of interest.
